# *Notes from the Field:* Measles Outbreak in an Era of Stricter Immunization Requirements — California, March 2018

**DOI:** 10.15585/mmwr.mm6808a3

**Published:** 2019-03-01

**Authors:** George Han, Neale Batra, Alvin Vallejo, Robert Schechter, Jennifer Zipprich, Kathleen Harriman

**Affiliations:** ^1^Santa Clara County Public Health Department, San Jose, California; ^2^California Epidemiologic Investigation Service; ^3^California Department of Public Health.

On March 4, 2018, an unvaccinated adolescent boy (patient A, aged 15 years) who had recently returned from England and Wales, where measles outbreaks were occurring, was evaluated by a physician for fever, cough, coryza, conjunctivitis, Koplik spots, and rash. Measles virus nucleic acid was detected in an oropharyngeal swab and in urine tested at the Santa Clara County (California) Public Health Department (SCCPHD). Nineteen days later, on March 23, measles was reported in an unvaccinated adolescent boy (patient B, aged 16 years) who had been at a scouting event with patient A (Figure). Patient B was not contacted during public health investigation because patient A had not reported attending this event. On March 24, an unvaccinated male classmate of patient A’s (patient C, aged 15 years) developed measles while in quarantine. On April 2, a man (patient D, aged 21 years) who had received 2 doses of measles, mumps, and rubella (MMR) vaccine and who had attended a different scouting event in Santa Clara County with patient B before returning to college in Nevada was reported as a measles patient to the Washoe County (Nevada) Health District.

**FIGURE:**
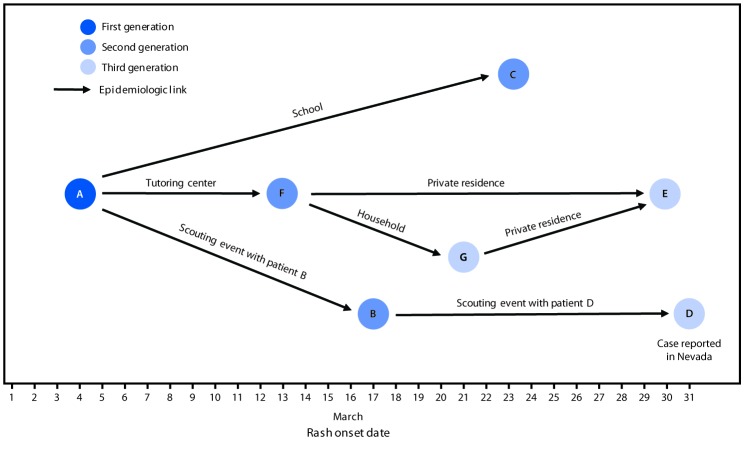
Measles transmission associated with community exposures to persons who had not received measles, mumps, and rubella vaccine, by date of rash onset — California, March 2018*^,†^ * Patients A–E had measles genotype D8. The parents of patients F and G did not consent to laboratory testing. ^†^ Patient E could have been infected by either patient F or patient G during a visit to their home on March 17.

On April 3, the Alameda County (California) Public Health Department received a report of measles in an unvaccinated man (patient E, aged 33 years). He identified his nephew (patient F, aged 7 years) as the source of his illness but declined to provide contact information. SCCPHD eventually confirmed his nephew’s presence at a tutoring center attended by patient A. The nephew’s parents could not be reached by phone; his mother was interviewed at their home. She acknowledged that her son was not vaccinated and revealed that both he and his unvaccinated brother (patient G, aged 4 years) had experienced recent illnesses consistent with measles. Hundreds of contacts of these seven patients were traced across 10 counties in California and Nevada.

Although patient A’s parents had chosen not to vaccinate him, his immunocompromised brother, an organ transplant recipient, had received intravenous immunoglobulin to protect him against measles before traveling overseas. When patient A’s illness was reported, SCCPHD recommended that his brother receive additional intravenous immunoglobulin and be quarantined 7 additional days; the family followed both recommendations. Patient C’s unvaccinated sister, aged 17 years, received parental permission to choose to receive MMR vaccine when her brother was quarantined; she opted to receive the vaccine. Patient D, who had received 2 doses of MMR vaccine, exhibited mild symptoms consistent with modified measles (*1*). None of his many contacts at a large university developed measles.

MMR vaccine is recommended for all persons born in the United States since 1957 who do not have a contraindication for the vaccine.* In this outbreak, the six unvaccinated patients with measles all had parents who had chosen not to vaccinate them during childhood. Since California Senate Bill 277 (SB277) went into effect in 2016, children entering school in California may no longer receive exemptions from immunization requirements based on parental personal beliefs.^†^ However, medical exemptions for reasons determined by individual physicians, including family medical history, rather than a uniform standard (i.e., a medical contraindication to vaccination), remain permitted (*2*). Interviews with local health authorities suggest that some students without contraindications to vaccination have received medical exemptions (*3*). Patients F and G received identical broad medical exemptions to all vaccines from a physician located several hundred miles away from the patients’ residence. Patients E and G represent the first documented cases of measles in California infected by a child with a medical exemption since SB277 became law; had SCCPHD received accurate information about patient F’s immunization status, these two illnesses might have been prevented, and the expenditure of resources to investigate their contacts might have been avoided. Prompt public health action and continued maintenance of a high level of population immunity to measles likely averted a larger outbreak.

## Footnotes

* https://www.cdc.gov/mmwr/preview/mmwrhtml/rr6204a1.htm.

† https://leginfo.legislature.ca.gov/faces/billNavClient.xhtml?bill_id=201520160SB277.
